# Quantification of cerebral veins in patients with acute migraine with aura: A fully automated quantification algorithm using susceptibility-weighted imaging

**DOI:** 10.1371/journal.pone.0233992

**Published:** 2020-06-03

**Authors:** Philipe Sebastian Breiding, Frauke Kellner-Weldon, Lorenz Grunder, Adrian Scutelnic, Urs Fischer, Thomas Raphael Meinel, Nedelina Slavova, Jan Gralla, Marwan El-Koussy, Niklaus Denier

**Affiliations:** 1 Institute of Diagnostic and Interventional Radiology, Cantonal Hospital Frauenfeld, Spital Thurgau AG, Frauenfeld, Switzerland; 2 Institute of Diagnostic and Interventional Radiology, Cantonal Hospital Luzern, Luzern, Switzerland; 3 Support Center for Advanced Neuroimaging (SCAN), University Institute of Diagnostic and Interventional Neuroradiology, University of Bern, Inselspital, Bern, Switzerland; 4 University Institute of Diagnostic and Interventional Radiology, University of Bern, Inselspital, Bern, Switzerland; 5 Department of Neurology, University of Bern, Inselspital, Bern, Switzerland; 6 University Institute of Diagnostic and Interventional Neuroradiology, University of Bern, Inselspital, Bern, Switzerland; 7 Department of Psychiatry and Psychotherapy, University of Bern, Bern, Switzerland; Universitatsklinikum Freiburg, GERMANY

## Abstract

**Introduction:**

Susceptibility weighted imaging (SWI) is a very sensitive technique that often depicts prominent focal veins (PFV) in patients with acute migraine with aura (MwA). Interpretation of visual venous asymmetry (VVA) between brain hemispheres on SWI may help support the clinical diagnosis of MwA. Our goal was to develop an automated algorithm for segmentation and quantification of cerebral veins using SWI.

**Materials and methods:**

Expert readers visually evaluated SWI of patients with acute MwA for VVA. Subsequently a fully automated algorithm based on 3D normalization and 2D imaging processing using SPM and MATLAB image processing software including top-hat transform was used to quantify cerebral veins and to calculate volumetric differences between hemispheres.

**Results:**

Fifty patients with MwA were examined with SWI. VVA was present in 20 of 50 patients (40%). In 95% of patients with VVA, the fully automated calculation agreed with the side that visually harboured more PFV. Our algorithm showed a sensitivity of 95%, specificity of 90% and accuracy of 92% for detecting VVA. Patients with VVA had significantly larger vein volume on the hemisphere with more PFV compared to patients without *(15*.*90 ± 5*.*38 ml vs 11*.*93 ± 5*.*31 ml; p = 0*.*013)*. The mean difference in venous volume between hemispheres in patients with VVA was larger compared to patients without VVA *(16*.*34 ± 7*.*76% vs 4*.*31 ± 3*.*26% p < 1E-10)*. The average time between aura onset and SWI correlated negatively with venous volume of the dominant brain hemisphere *(r = -0*.*348; p = 0*.*038)*.

**Conclusion:**

A fully automated algorithm can accurately identify and quantify cerebral venous distribution on SWI. Absolute quantification may be useful for the future assessment of patients with suspected diseases, which may be associated with a unilateral abnormal degree of venous oxygenation.

## Introduction

Susceptibility weighted imaging (SWI) is a widely used magnetic resonance imaging (MRI) technique that utilizes the magnetic field inhomogeneity of deoxygenated haemoglobin and subsequent reduction of T2* weighted signal to visualize the cerebral venous system [1]. It is a very sensitive technique that has proven to be beneficial in the diagnosis of a variety of intracranial pathologies including vascular malformations, intracranial haemorrhage, neoplasms and multiple sclerosis [2, 3].

A feature that distinguishes SWI from other MRI sequences is high-resolution demarcation of cerebral venous architecture, which can be visually enhanced by minimum intensity projection (MinIP). This technique enables neuroradiologists to gain an accurate overview of the cortical and subcortical venous distribution of the brain and to make a visual judgement as to whether the venous distribution is symmetrical or asymmetrical. Studies have shown that SWI could reveal asymmetry of the cortical venous vasculature in specific regions of the brain following ischemic stroke, epilepsy and migraine [4–6]. Patients who undergo imaging during a migraine-associated aura characteristically display unilateral brain hypoperfusion and subsequent prominent venous vasculature on SWI [7]. Frequently these findings are only very discreet and may go unnoticed on initial image evaluation which is why a computer-assisted approach may aid in making the correct diagnosis.

Our goal was to create a fully automated algorithm for the quantification of cerebral veins, which could identify hemispherical venous asymmetry using patients with a clinically proven episode of acute migraine with aura (MwA).

## Methods

### Study cohort

From a prospectively maintained clinical database, we retrospectively identified and analysed all patients who were diagnosed with an acute episode of MwA in an emergency setting between 01/2014 and 05/2018. Inclusion criteria were a) confirmed MwA according to the International Classification of Headache Disorders [8], confirmed by a board-certified neurologist either at discharge or at follow-up visits, b) 1.5-Tesla brain MRI within 24-hours of symptom onset with acquisition of SWI and reconstruction of MinIP and c) exclusion of stroke, tumor, haemorrhage or sinus vein thrombosis.

Clinical and baseline characteristics of patients are summarized in Table 1. This study received approval from the local ethics committee (Ethics Commission of the Canton of Bern, Bern, Switzerland, Project ID: PB_2018–00128). The ethics committee waived the requirement for informed consent as the data were fully anonymized before being accessed.

**Table 1:** Baseline characteristics of 50 patients with acute migraine with aura who received a 1.5T MRI.

**Acute migraine with aura: No. (%)**			
**Variable**	**No VVA (n = 30)**	**VVA (n = 20)**	***p* Value**
Age *(years)* ^a^	36.90 ± 14.18	38.70 ± 15.61	0.676
Female	19 (63.33)	12 (60.0)	0.812
Positive family history of migraine	7 (23.33)	6 (30.0)	0.852
Left-sided VDH	N/A	15 (75.0)	-
DCVV (*ml*) ^a^	1.18 ± 1.17	4.56 ± 2.16	< 1E-10 ***
*n*DCVV *(%)* ^a^	4.31 ± 3.26	16.34 ± 7.76	< 1E-10 ***
CDH Volume *(ml)* ^a^	11.93 ± 5.31	15.90 ± 5.38	0.013 *
CNDH Volume *(ml)* ^a^	10.75 ± 4.60	11.34 ± 4.42	0.66
**Time interval btw. aura onset and MRI *(hours*: *min)*** ^a^ **(n = 36)**			
	**No VVA (n = 22)**	**VVA (n = 14)**	***p* Value**
	05:09 ± 02:25	03:58 ± 01:56	0.132

CDH: Calculated Dominant Hemisphere, CNDH: Calculated Non-dominant Hemisphere, DCVV: Difference in absolute Cerebral Vein Volume, *n*DCVV: Normalised Difference in absolute Cerebral Vein Volume, VDH: Visually Dominant Hemisphere, VVA: Visual Venous Asymmetry, N/A: Not available.

* Significant for p < 0.05.

*** Significant for p < 1E-10.

^a^ Values presented as mean ± SD

### Image acquisition

All imaging studies were performed using a 1.5-Tesla Siemens scanner (Magnetom Avanto or Magnetom Aera; Siemens Medical Solution, Erlangen, Germany) with a 12-channel head coil. The following SWI parameters were used: TR 49 ms, TE 40 ms, number of averages 1, FoV read 230 mm, FoV phase 81.3%, voxel size 1.1 × 0.9 × 1.8 mm, FA 15°. Slice thickness of MinIP ranged between 12.8 and 16.0 mm (14.21 ± 0.99). SWI images were calculated by the scanner software using magnitude and high pass filtered phase images. Additionally, diffusion- (DWI) and perfusion-weighted imaging was performed to rule out acute stroke in an emergency setting. DWI sequence parameters for 1.5 Tesla: TR 3000 ms, TE 89 ms, number of averages 4, FOV read 230 mm, FOV phase 100%, voxel size 1.2 mm × 1.2 mm × 5.0 mm.

### Image analysis by readers

DWI images were read by one board-certified neuroradiologist (Frauke Kellner-Weldon, MD 16 years of experience). SWI images were assessed collectively by one board-certified neuroradiologist (Frauke Kellner-Weldon, MD 16 years of experience) and two Neuroradiology fellows (Philipe Breiding, MD and Lorenz Grunder, MD each with 2 years of experience). The readers were blinded to patient clinical data and all imaging studies performed on a different date. The images were reviewed on a picture archiving and communications system (PACS) station (R11·4·1, 2009; Philips, Best, Netherlands; Sectra, Linkoping, Sweden). Only SWI and MinIP sequences were previously arranged and stored on an established, non-modifiable layout.

**Fig 1:**
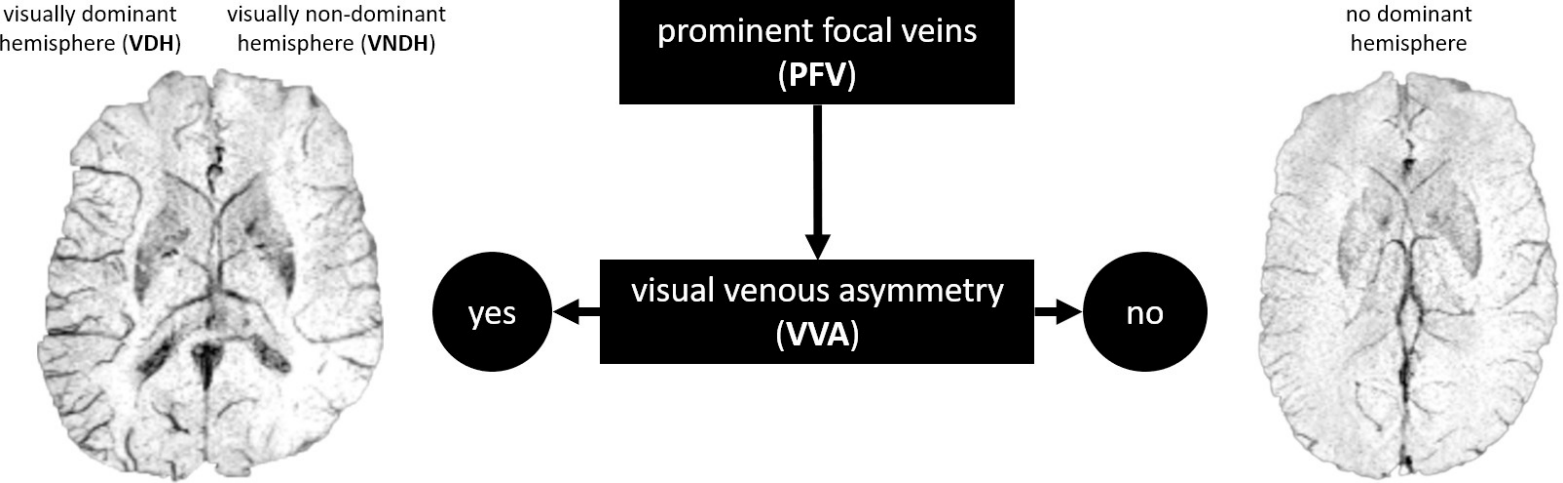
Image analysis procedure by readers. SWI MinIP of two separate patients with acute migraine with aura. Left: visual venous asymmetry (VVA) with radiological right-sided visually dominant hemisphere (VDH). Right: no VVA and no VDH.

The readers reviewed SWI and MinIP images for each patient and distinguished whether visual venous asymmetry (VVA) between brain hemispheres was absent or present (yes or no). VVA was defined as present if a single brain hemisphere showed more prominent focal veins (PFV) than the contralateral hemisphere. If VVA was present, the readers were asked to denote the hemisphere which harboured most PFV as the pathological side. This side was defined as the visually dominant hemisphere (VDH) and the contralateral hemisphere was defined as the visually non-dominant hemisphere (VNDH) respectively (Fig 1).

Only patients for whom there was a consensus amongst all three readers were included into the study and these patients were subsequently divided into two groups: (1) Patients with VVA and (2) patients without VVA.

A total of 50 patients who received a 1.5-Tesla MRI with SWI and MinIP images of sufficient quality within 24-hours of aura onset were included into our study.

### Automated quantification of cortical and subcortical veins

**Fig 2:**
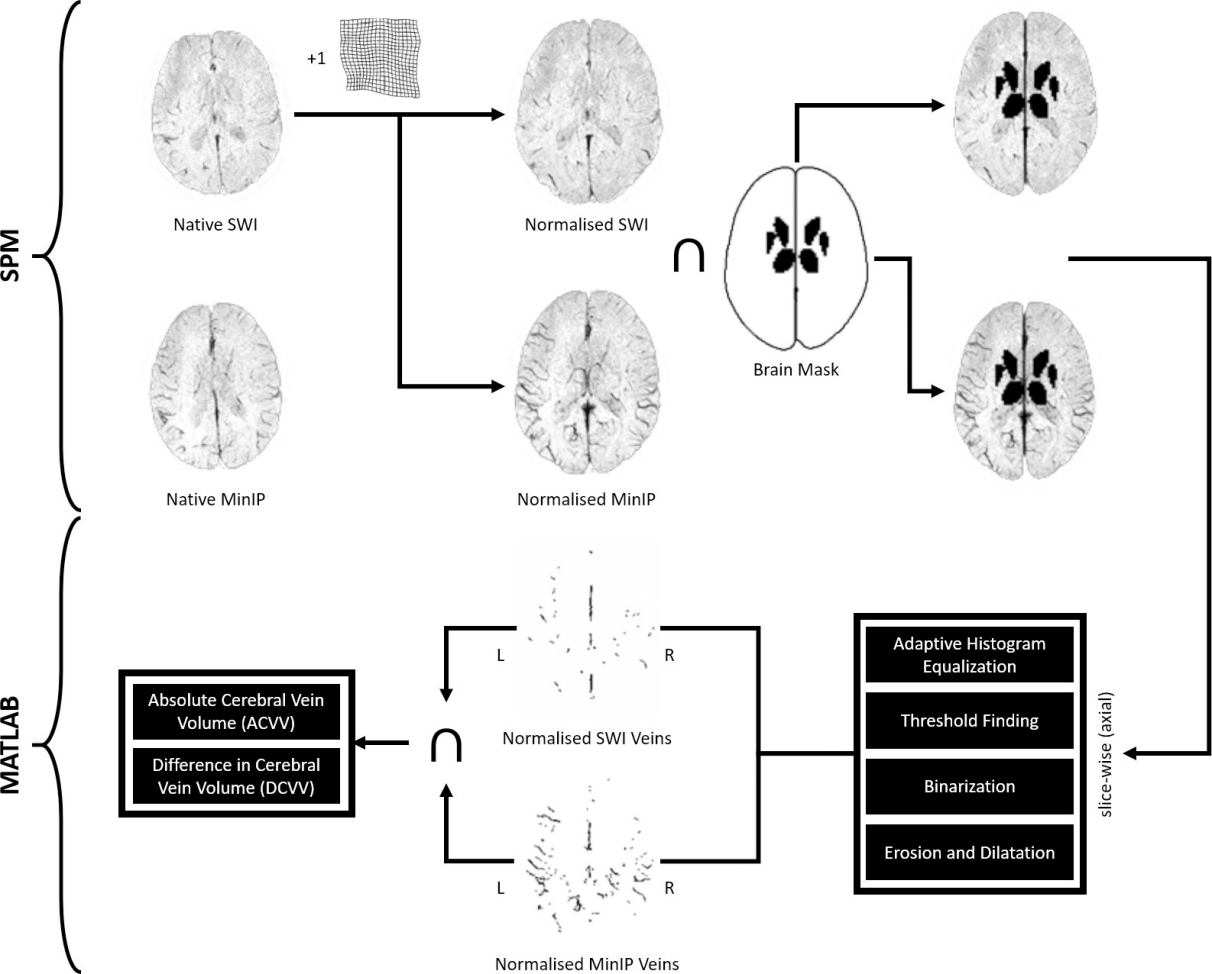
Processing pipeline of automated quantification of cortical and subcortical veins.

**Fig 3:**
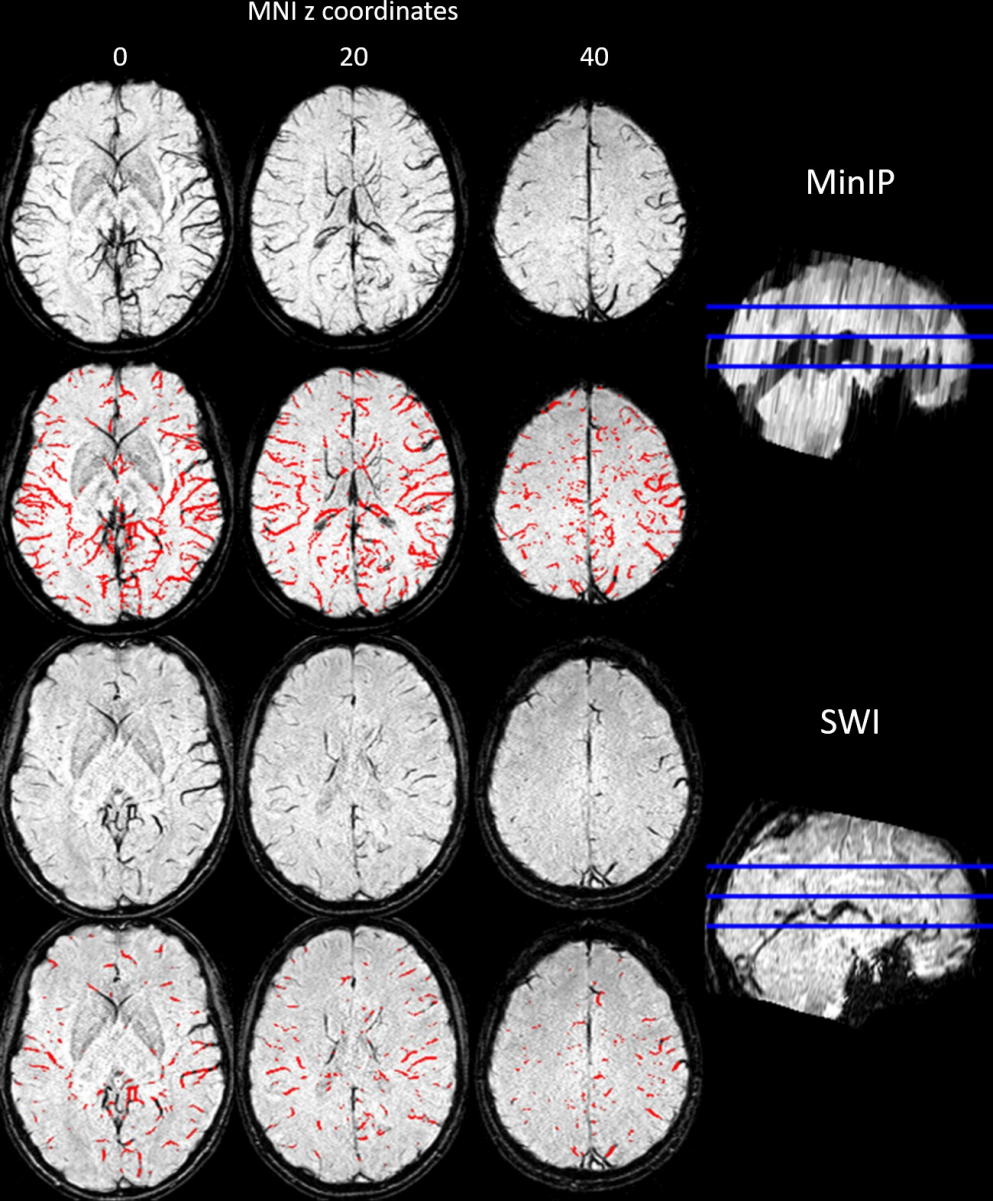
Example of extracted veins in normalised MinIP and SWI. Final vein extraction is shown in the red SWI overlay with a slice thickness of 1 mm.

SWI and MinIP images were postprocessed using MATLAB (R2018) and SPM 12 (http://www.fil.ion.ucl.ac.uk/spm/software/spm12) (Fig 2). Native SWI images were normalised into Montreal Neurological Institute (MNI) space using a T1 template. The resulting transformation matrix were applied to the MinIP images. The normalized SWI and MinIP images were masked with a binary brain mask with exclusion of the thalami and the basal ganglia due to low SWI signal intensity. After normalisation and masking, the resulting SWI and MinIP images were separately analysed slice wise (axial) using MATLAB image toolbox functions with a customized script. Grey value contrast of SWI and MinIP images were enhanced and normalised using the *adapthisteq* function by transforming the grey values using contrast-limited adaptive histogram equalization [9]. Optimal threshold for binarization of each SWI and MinIP slice was found using the Otsu’s threshold selection method implemented in the function *graytresh* [10]. The resulting SWI and MinIP images were inverted (veins white) and binarized using the *im2bw* function. Morphological top-hat transform, implemented as *bwmorp* function with *tophat* operation, were used for further vein extraction in SWI and MinIP images by calculating the difference between the input image and its morphological opening (erosion followed by dilation) through a standard structuring element [11, 12]. The top-hat transform was important for removal of the inverted background and of noise. After slice-wise pre-processing with top-hat transforms, the resulting SWI (*I*_1_) and MinIP (*I*_2_) images were used to further enhance segmentation of cortical and subcortical veins (*V*) for each hemisphere (*H*) by calculating their intersect: *V* = *I*_1_∩*I*_2_∩*H* (Fig 3). Finally, absolute cerebral vein volume (ACVV) in ml was calculated for each individual hemisphere over all axial slices. The hemisphere with a higher ACVV was defined as the calculated dominant hemisphere (CDH) and the hemisphere with a lower ACVV as the calculated non-dominant hemisphere (CNDH). The difference in absolute cerebral vein volume between brain hemispheres (DCVV) was expressed in ml by subtracting the CNDH volume from the CDH volume: *DCVV = CDH−CNDH*. The normalized DCVV (*n*DCVV) was calculated by dividing the DCVV through the sum of the dominant and non-dominant hemispheres: $nDCVV=\frac{DCVV}{CDH+CNDH}\times 100\%.$

### Statistical analysis

Statistical comparisons of demographic, clinical and imaging characteristics were performed with SPSS 25 (IBM SPSS Statistics; Armonk, NY: IBM Corp) using independent sample t-tests for normal distributed parametric and χ^2^ tests for nonparametric data. For bivariate correlations, we used Pearson’s correlation coefficient. We calculated the sensitivity, specificity and accuracy of our algorithm to detect VVA after finding an optimal DCVV cut-off using Receiver Operating Characteristics (ROC) and Youden-Index (J). A ROC curve was generated using SPSS. For all statistical analyses, the level of significance was set at p < 0.05 (two-tailed).

## Results

A total of 50 patients with acute migraine with aura (*31 females*, *62%*) were included into our study (*mean age 37*.*62 y [14–80 ± 14*.*64*]). Family history of migraine was positive in 26%, negative in 28% and unknown in 46% of patients. The average time between aura onset and MRI was 4h 41 min ± 2h 17 min (*min*: *1h 22min*, *max*: *10h 13min*). In addition to aura, headache symptoms were recorded for a total of 41 patients upon presentation to the emergency room. 68% of these patients expressed unilateral headache, 12% bilateral headache and 20% no headache (Table 2).

**Table 2:** Crosstabs table of 41 patients for whom headache symptoms were recorded upon presentation to the emergency room. Values represent number of patients.

		Visually Dominant Hemisphere			
**Headache Side**		**None**	**Right**	**Left**	**Total (n = 41)**
	**None**	5	1	2	8
	**Right**	4	4	0	8
	**Left**	15	0	5	20
	**Both**	3	0	2	5

The readers noted VVA in 20 of 50 (40%) of patients and 75% of these patients showed a VDH on the left side (Table 1). No patients showed any focal areas of diffusion restriction on DWI as a possible sign of acute stroke and SWI did not reveal any patients with micro- or macrohaemorrhage.

**Fig 4:**
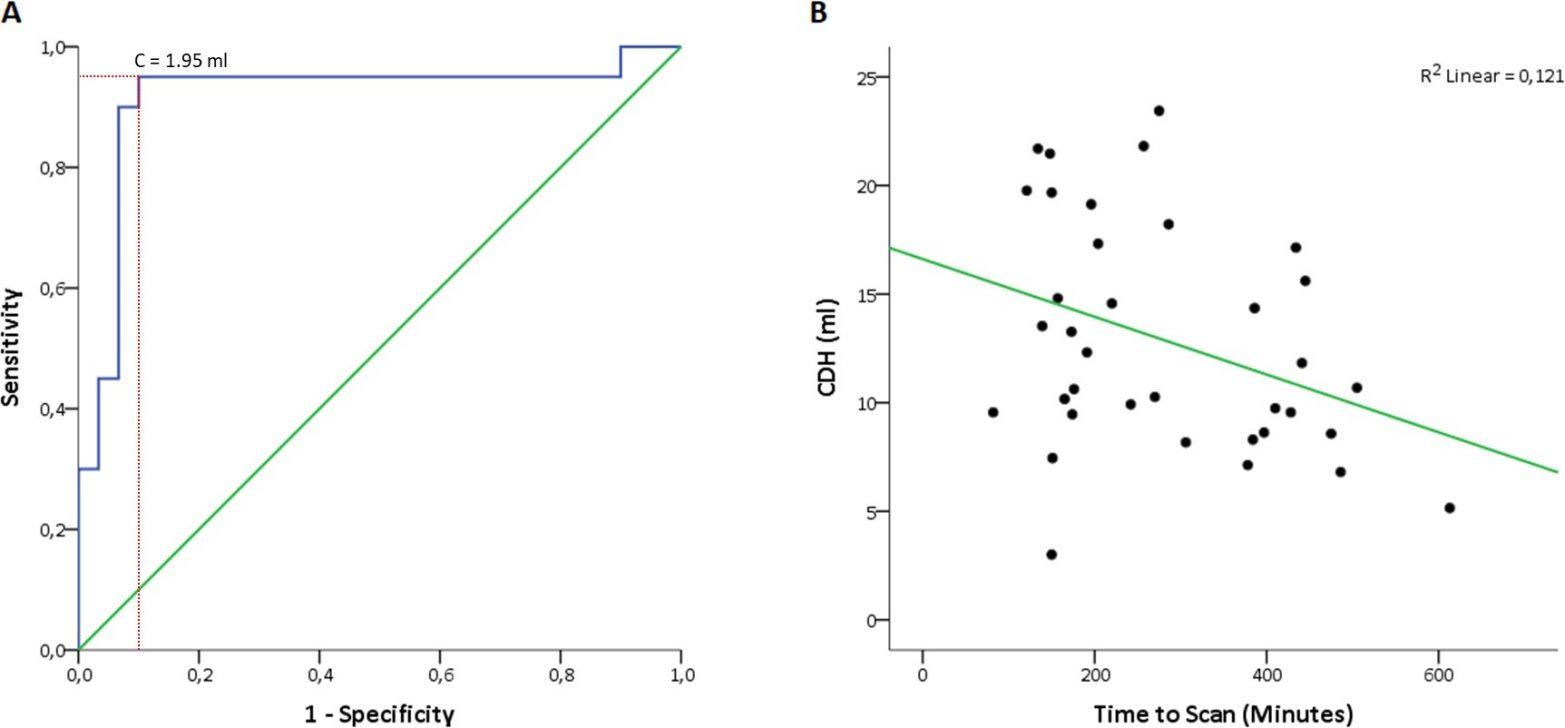
A) ROC curve showing the ability of our automated algorithm to discriminate between VVA and no VVA. B) Scatter plot graph depicting a negative correlation between time to scan and CDH volume.

All SWI and MinIP images were successfully imported and processed by our algorithm. Our algorithm showed a sensitivity of 95%, specificity of 90% and accuracy of 92% for detecting VVA using a DCVV cut-off value of 1.95 ml (Fig 4). In 19 of 20 patients with VVA (95%), the CDH side was the same as the VDH side.

Patients with VVA had a significantly larger CDH volume than patients without VVA *(15*.*90 ± 5*.*38 ml vs 11*.*93 ± 5*.*31 ml; p = 0*.*013)*. The *n*DCVV was significantly larger in patients with VVA compared to patients without VVA *(16*.*34 ± 7*.*76% vs 4*.*31 ± 3*.*26%; p < 1E-10)*. There were no significant differences in CNDH volume between patients with VVA and patients without VVA *(11*.*34 ± 4*.*42 ml vs 10*.*75 ± 4*.*60 ml; p = 0*.*66)*.

The average time between aura onset and MRI correlated negatively with the CDH volume for n = 36 *(r = -0*.*348; p = 0*.*038)*. Patient age did not correlate significantly with the CDH volume *(r = 0*.*207; p = 0*.*15)* and did not correlate with the *n*DCVV *(r = 0*.*017; p = 0*.*91)*. Headache side was significantly associated with the VDH for n = 41 *(χ*^*2*^ *= 15*.*64; p = 0*.*016)*.

## Discussion

Using a fully automated computer algorithm, we were able to quantify cerebral veins for individual brain hemispheres using all SWI and MinIP sequences obtained from 50 patients who presented to the emergency room with an acute migraine with aura attack and who received 1.5-Tesla MR-imaging within 24-hours of symptom onset.

Our findings show multiple advantages of an automated computer algorithm. The algorithm was firstly able to quantify the absolute venous volume of individual brain hemispheres using readily available SWI and MinIP sequences. Secondly, show that there is a quantifiable difference in venous volume between brain hemispheres in patients with acute migraine with aura. Thirdly, it was able to accurately identify the respective brain hemisphere which harboured more prominent focal veins in patients with visual venous asymmetry between brain hemispheres.

In our cohort of patients with MwA we found that in 20 of 50 patients, SWI would show visual asymmetry between hemispheres. Migraine with aura may clinically present with acute deficits mimicking acute ischemic stroke. However, the signal changes, which we observed on SWI, did not follow a territorial vascular distribution and DWI was normal. Both these findings helped to exclude stroke.

Only few studies have thus far dealt with the quantification of cerebral veins using SWI. One fully automated SWI quantification algorithm has been described in literature by Egger et. al. who developed a voxel-based algorithm using SPM8 and Advanced Normalization Tools (ANTs) to quantify the absolute cerebral vein volume in ten healthy individuals [16]. Dempfle et. al. subsequently used this algorithm to quantify deep medullary veins in patients with cerebral venous sinus thrombosis [17]. Due to the fact that these studies excluded cortical veins in the quantification process and did not focus on individual brain hemispheres, it remains difficult to compare our results to these studies. To the best of our knowledge, automated quantification of cerebral veins in individual brain hemispheres with inclusion of cortical veins has thus far not been described. Our vein segmentation algorithm was applied to SWI data, however, quantitative susceptilility mapping (QSM) may be useful by its quantitative measurement for venous vessel segmentation [18]. Ward et al. could show that fusion of QSM and SWI for automated segmentation resulted in a more robust segmentation of cortical and subcortical veins [19]. In general, the use of several parameters seems to be advantageous for vein segmentation. Monti et al. achieved good venous segmentation results using a multi-parametric approach (structural, morphological and relaxiometric information) [20]. Overall, QSM shows major advantages for venous segmentation, but is not readily implemented in clinical routine.

We found a negative correlation between time elapsed and the CDH volume. The most likely explanation for this is the time-dependent decrease of deoxygenation of venous cerebral blood throughout the course of MwA, with decreasing conspicuity of the venous vasculature on SWI over time [13, 14].

Patient age correlated positively with the CDH volume but this correlation was not significant. We do not know of any study that has investigated age depended differences of oxygenation fraction (OEF) in venous blood in MwA. However, we think that in clinical practice, we need to be aware of age depended differences of brain perfusion and OEF in healthy individuals, which might contribute to differences in OEF in MwA patients [15].

We found a significant association between headache side and the brain hemisphere that visually harboured more PFV. Previous studies have shown that migraine with aura is often associated with unilateral changes in cerebral perfusion which may provoke headache symptoms [7, 13]. We believe that the brain hemisphere most affected by these hemodynamic changes on average show more PFV [21] and may therefore represent the clinically affected side.

Our study is limited by the cohort size, which is mainly due to the strict inclusion and exclusion criteria that we implemented. We insisted on using the same Tesla strength for this feasibility study. The algorithm needs yet to be tested in more clinical circumstances, where MRI machines of different field strength and vendors are likely to be used.

Another limitation is the presence of magnetic field inhomogeneity artefacts at the cortical border or other conditions such as microbleeds and calcifications which appear hypointense on SWI and which were most likely included into the quantification by our automated algorithm as well. Nonetheless, prior to quantification we made sure that all images were of sufficient quality and no patients showed any signs of micro- / macrohemorrhage on SWI. The results show that the algorithm is potentially robust enough to be of use even under these circumstances.

A limitation is that we could not compare our quantified volumes to a healthy volunteer group and that automated volumetric data for cerebral veins currently does not exist in literature. The quantified absolute cerebral vein volumes in individual brain hemispheres showed a large range from 3.00 to 24.85 ml, which may be attributable to the presence of above-mentioned artefacts or due to differences in cerebral vein anatomy between individual patients. These differences may stem from blood oxygenation status [21, 22], caffeine intake [23], haematocrit [17], and other factors that may influence the T2*-weighted signal of venous blood on SWI. Nevertheless, we aimed at simulating a clinical scenario, where these factors are routinely not readily modifiable, when emergency MR imaging is performed.

A disadvantage of the automatic approach is the additional time required for computation. The clinical interpretation of whether venous asymmetry is present only takes seconds. However, a quantitative approach can be particularly interesting in the context of studies, as it is a more objective approach. The identification of venous asymmetry can support the clinical diagnosis of a migraine but not diagnose it. Asymmetry can also occur in other pathologies, such as epilepsy or stroke. Independent of pathologies, further studies are needed to investigate the intraindividual characteristics of SWI neuroimaging.

## Conclusions

In patients with MwA, an automated computer algorithm can identify and quantify cerebral veins in individual brain hemispheres and accurately determine the respective brain hemisphere that harbours more prominent focal veins. Volumetry of cerebral veins seems to be a valuable tool for the future assessment of patients with suspected diseases, which may be associated with a unilateral abnormal degree of venous oxygenation. SWI in addition to DWI was useful in the assessment patients with MwA, to rule out significant differential diagnoses, such as stroke.

## Supporting information

S1 Table (XLS)

## Abbreviations

ACVV: Absolute Cerebral Vein Volume [ml]
CDH: Calculated Dominant Hemisphere (dominant ACVV) [ml]
CNDH: Calculated Non-Dominant Hemisphere (non-dominant ACVV) [ml
DCVV: Difference in absolute Cerebral Vein Volume (*CDH−CNDH*) [ml
*n*DCVV: Normalised Difference in absolute Cerebral Vein Volume ($\frac{DCVV}{CDH+CNDH}\times 100$) [%]
DWI: Diffusion-weighted Imaging
MinIP: Minimum Intensity Projection
MRI: Magnetic Resonance Imaging
MwA: Migraine with Aura
OEF: Oxygenation Fraction
PACS: Picture Archiving and Communications System
PFV: Prominent Focal Veins
ROC: Receiver Operating Characteristics
SWI: Susceptibility-Weighted Imaging
VDH: Visually Dominant Hemisphere
VNDH: Visually Non-Dominant Hemisphere
VVA: Visual Venous Asymmetry
